# Effect of Hyaluronic Acid Molecular Weight on Physicochemical and Rheological Properties of Ice Cream

**DOI:** 10.1002/fsn3.72444

**Published:** 2026-10-03

**Authors:** Rutvi Joshi, Mahmoud E. A. Hamouda, Prafulla Salunke

**Affiliations:** ^1^ Dairy and Food Science Department, Midwest Dairy Foods and Research Center South Dakota State University Brookings South Dakota USA

**Keywords:** hyaluronic acid, ice cream, molecular weight, rheology, stabilizers, texture profile

## Abstract

This study evaluated the effect of hyaluronic acid (HA) with different molecular weights on the physicochemical and rheological properties of ice cream. Ice cream formulations were prepared using a consistent base mix, with treatments including a control sample containing 0.3% commercial stabilizer–emulsifier blend (IC‐C) and 4 HA‐treated samples with 0.3% HA at molecular weights of 320 kDa (IC‐1), 980 kDa (IC‐2), 1550 kDa (IC‐3), and 2550 kDa (IC‐4). Analyses were performed on both the ice cream mix and final ice cream to assess functional properties. Overrun, hardness, adhesiveness, and melting rate were evaluated in ice cream samples, while creaming stability (expressed as creaming index) and rheological properties (G′, G″, and viscosity) were assessed in the mix. The experiment was replicated three times. Results showed that HA treatments had no significant effect (*p* > 0.05) on overrun. However, HA significantly influenced textural and rheological properties depending on molecular weight. IC‐3 and IC‐4 exhibited significantly higher (*p* < 0.05) hardness and lower (*p* < 0.05) melting rates compared to IC‐1 and the control. Creaming stability improved with increasing HA molecular weight, as indicated by lower creaming index values over 21 days. Rheological analysis showed increases in storage modulus, loss modulus, and viscosity with increasing HA molecular weight. Overall, higher molecular weight HA (1550 and 2550 kDa) improved several measured ice cream properties under the conditions of this study and shows potential as a structuring ingredient. However, as the control formulation contained both stabilizers and emulsifiers, these results do not demonstrate full replacement of conventional stabilizer–emulsifier systems.

## Graphical abstract

We prepared ice cream formulations, including a control with a commercial stabilizer–emulsifier blend at 0.3% and four treatments with 0.3% hyaluronic acid (HA) at different molecular weights (320, 980, 1550, and 2550 kDa), and compared them. Increasing HA molecular weight was associated with increased hardness, improved creaming stability, enhanced rheological properties, and a reduced melting rate. Higher HA molecular weights, particularly 1550 and 2550 kDa, had the greatest impact on the measured physicochemical and rheological properties.

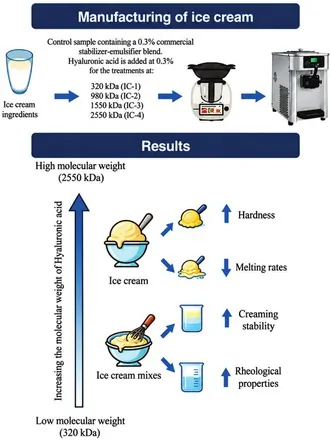

## 1 Introduction

Ice cream is one of the most popular dairy desserts worldwide, valued for its smooth texture, rich flavor, and refreshing appeal (Weiss 2012). Despite its widespread consumption, manufacturing high‐quality ice cream presents several technical challenges. Among these, maintaining a stable structure, desirable texture, and consistent sensory properties over shelf life remains difficult (Marshall et al. 2003). This is primarily due to the complex nature of the product, which is a partially frozen emulsion containing air cells, fat globules, ice crystals, and a continuous aqueous phase (Goff 1997). The formulation must therefore be carefully designed to control ice recrystallization, melting behavior, and structural stability during freezing, storage, and distribution. To address these challenges, polysaccharide‐based stabilizers are commonly incorporated into ice cream formulations to control moisture migration, inhibit ice recrystallization, and enhance overall texture (Giridharaprasad et al. 2024). Widely used stabilizers, such as guar gum, carrageenan, locust bean gum, and carboxymethyl cellulose (CMC), have been shown to improve viscosity and freeze–thaw stability (Maity et al. 2018). However, depending on their concentration and interactions with other ingredients, these stabilizers may contribute to undesirable textural attributes such as excessive gumminess. This has led to increasing interest in alternative polysaccharides that can provide improved functionality while maintaining desirable sensory properties. Hyaluronic acid (HA) has been investigated as a potential functional ingredient in food systems due to its high water‐binding capacity. Its ability to retain water contributes to increased viscosity, improved texture, and enhanced stability in various food matrices. In dairy products such as milk (Joshi et al. 2024; Sutariya and Salunke 2022, 2023) and processed cheese (Joshi et al. 2025), HA has been reported to enhance water‐holding capacity and influence structural properties. Previous studies (Joshi et al. 2024; Sutariya and Salunke 2022) have also indicated that HA may improve emulsion stability and protein interactions in dairy systems, which may be relevant to frozen dairy desserts such as ice cream. HA is a naturally occurring linear polysaccharide composed of repeating disaccharide units of β‐1,4‐D‐glucuronic acid and β‐1,3‐N‐acetyl‐D‐glucosamine (Gu et al. 2013). Its structure imparts a high negative charge density, enabling it to bind large amounts of water and form viscous systems (Xu et al. 2012). Another important characteristic of HA is that its functionality depends on molecular weight (MW) (Simińska‐Stanny et al. 2024; Joshi et al. 2024, 2025). Commercial HA is available across a wide MW range, from a few kilo‐Daltons to over 3000 kDa, with higher MW HA generally associated with increased viscosity and more pronounced viscoelastic behavior, while lower MW HA provides limited structural contribution (Bayer 2020). Currently, the US Food and Drug Administration (FDA) has not approved HA as a general food additive across all food categories. However, HA has been approved for use in food and functional products in several other regions, including Japan, South Korea, China, and parts of the European Union (Sutariya and Salunke 2022, 2023). Although HA has been previously explored in various dairy applications, limited information is available regarding the effect of its molecular weight on the physicochemical and rheological properties of ice cream. Therefore, this study aimed to evaluate the effects of varying HA molecular weights on the physicochemical, rheological, and textural properties of ice cream when used as a stabilizing ingredient, rather than to directly demonstrate the full replacement of conventional stabilizer–emulsifier systems.

## 2 Materials and Methods

### 2.1 Experimental Design

The study aimed to evaluate the effects of different MWs of HA (320kDa, 980 kDa, 1550 kDa, and 2550 kDa) in the production of ice cream, substituting traditional stabilizers. The treatments were applied to separate batches of ice cream, each incorporating a specific MW of HA. The control was made using the standard stabilizer blend. The experimental design, including the various treatments and ice cream mix (ICM) and ice cream (IC) codes, is outlined in Table 1.

**TABLE 1:** Ice cream mix (ICM) and ice cream (IC) sample codes for treatments with varying stabilizer and hyaluronic acid (HA).

Ice cream mix sample code	Ice cream sample code	Commercial stabilizer %	HA %
ICM‐C	IC‐C	0.3	—
ICM‐1	IC‐1	—	0.3 (320 kDa)
ICM‐2	IC‐2	—	0.3 (980 kDa)
ICM‐3	IC‐3	—	0.3 (1550 kDa)
ICM‐4	IC‐4	—	0.3 (2550 kDa)

### 2.2 Ice Cream Formulation

The ice cream formulation used in this study comprised a base mixture containing 33.92% cream (sourced from the Dairy Plant at South Dakota State University), 41.8% skim milk (obtained from the local market), 5.89% non‐fat dry milk (NFDM) (Continental Dairy Products, USA), 14.8% sugar (high‐grade, local market), and 3.29% dry corn syrup. In the control batch, 0.3% commercial stabilizer (CC392 from Tate & Lyle, IL, USA; mixture of guar gum, mono‐ and diglycerides, locust bean gum, carrageenan, and polysorbate 80 standardized with dextrose) was incorporated, whereas in the treatment batches, hyaluronic acid (HA; Haworks, China) was used as a substitute at the same concentration (0.3%). Batches were prepared to yield a total of 2000 g of ice cream (Table 2). The formulation resulted in an approximate composition of 12.2% fat, 4.1% protein, 11.2% milk solids‐not‐fat (MSNF), and 41.8% total solids, calculated based on standard composition values of the ingredients. It should be noted that the control formulation contained a commercial stabilizer–emulsifier blend (including mono‐ and diglycerides and polysorbate 80), whereas the HA treatments were prepared without added emulsifiers. Therefore, the comparison between control and HA treatments reflects differences in both stabilizer type and the presence of emulsifiers, which may influence the functional and structural properties of the ice cream systems.

**TABLE 2:** Ice cream mix formulation for control and treatment batches. Values are expressed as ingredient percentages (%), with the corresponding quantities for a 2000 g batch shown in the final column.

Ingredient	IC‐C	IC‐1	IC‐2	IC‐3	IC‐4	Batch size, 2000 g
Cream	33.92	33.92	33.92	33.92	33.92	678.4
Skim milk	41.8	41.8	41.8	41.8	41.8	836
NFDM	5.89	5.89	5.89	5.89	5.89	117.8
Sugar	14.8	14.8	14.8	14.8	14.8	296
Dry corn syrup	3.29	3.29	3.29	3.29	3.29	65.8
Stabilizer/emulsifier	0.3	—	—	—	—	6
Hyaluronic acid	—	0.3 (320 kDa)	0.3 (980 kDa)	0.3 (1550 kDa)	0.3 (2550 kDa)	6
Total	100	100	100	100	100	2000

Abbreviation: NFDM, non‐fat dry milk.

## 3 Methods

### 3.1 Ice Cream Mix and Ice Cream Manufacturing

A total of 2000 g of ingredients was weighed and blended in a thermomixer (TM6‐5, Vorwerk Elektrowerke GmbH & Co., Germany) with a 2 kg capacity at room temperature. The mixture was blended for approximately 10 min at a speed setting of 3 to achieve a homogeneous dispersion. During the final 60 s, the mixing speed was increased to 5, and hyaluronic acid (HA) was incorporated. No separate homogenization step was applied beyond thermomixing. The mix was pasteurized to 85°C for 20 s. The prepared ice cream mix was transferred into plastic containers and aged at 4°C overnight (~12–16 h). After aging, the mix was processed in a batch ice cream machine (Vevor, China) for 15 min to produce ice cream. The prepared ice cream was then transferred into plastic containers and hardened at −20°C for at least 24 h prior to subsequent texture and melting analyses (Figure 1).

**FIGURE 1:**
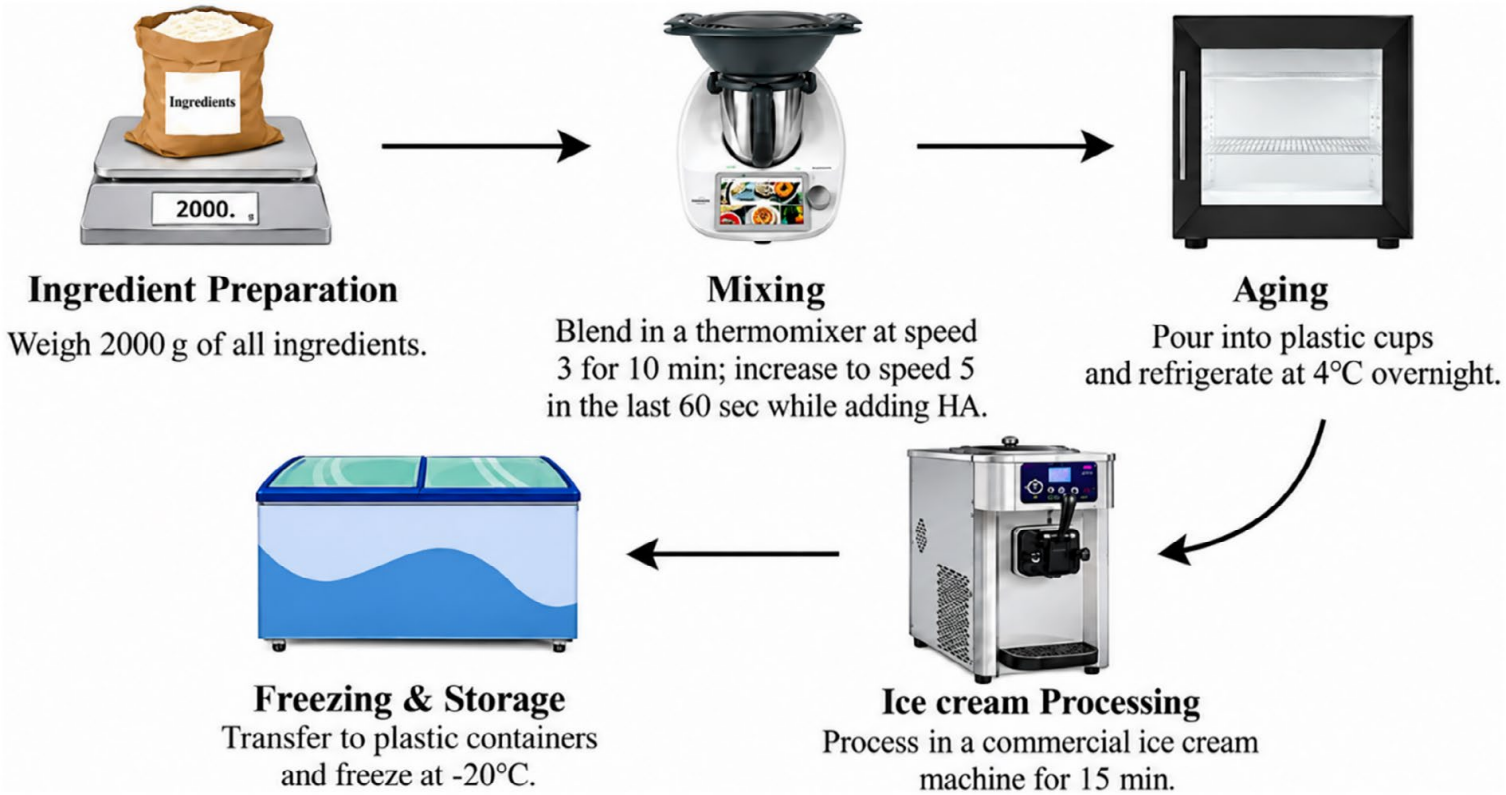
Ice cream mix and ice cream manufacturing.

#### 3.1.1 Ice Cream Mix Analysis

##### 3.1.1.1 Determination of Creaming Stability in Ice Cream Mix

Creaming stability was determined according to Cheng et al. (2015). A 50 mL sample of ice cream mix was transferred into centrifuge tubes, which were then stored at 4°C for a duration of 21 days. At 7 day intervals, the extent of phase separation was assessed, and the creaming stability was calculated using a predefined equation. The phase separation kinetics was determined as the creaming index (CI).

(1) $$\mathrm{CI}={\left(\mathrm{Hs}/\mathrm{He}\right)}^{*}100$$

where Hs is the height of the serum layer, and He is the total height of the emulsion.

##### 3.1.1.2 Rheological Characteristics

The rheological characteristics of the ice cream mix samples were measured using an Anton Paar rheometer (MCR 92, Anton Paar, Graz, Austria) equipped with a 25 mm parallel plate geometry (Sutariya and Salunke 2022). Approximately 2 g of sample was loaded, with a fixed gap of 1 mm. Samples were equilibrated at 4°C for 5 min prior to measurement to ensure temperature stability. All rheological measurements were subsequently performed at 4°C, with the instrument temperature maintained throughout the test. The storage modulus (G′), representing elastic behavior, and the loss modulus (G″), representing viscous behavior, were measured at a constant shear strain of 0.5% over an angular frequency range of 0.1 to 62.8 rad/s. The selected strain was within the linear viscoelastic region based on the literature (Sutariya and Salunke 2022). Data points were recorded at 5 rad/s intervals. Apparent viscosity (mPa·s) was measured using the same instrument over a shear rate range of 0.1 to 200 s^−1^, with measurements recorded every 5 s.

#### 3.1.2 Ice Cream Analysis

##### 3.1.2.1 Overrun

The overrun of each sample was determined as described by (Sofjan and Hartel 2004) by measuring the weight of the ice cream mix and the final ice cream in a fixed‐volume container. The overrun percentage was then calculated using the following formula:

$$\begin{array}{c} \text{Overrun}\;\left(\%\right)=\\ \left[\left(\text{Weight of}\;\mathrm{ice}\;\text{cream}\;\mathrm{mix}-\text{Weight of}\;\mathrm{ice}\;\text{cream}\right)/\text{Weight of}\;\mathrm{ice}\;\text{cream}\right]\times 100 \end{array}$$

where both weights are measured using the same fixed‐volume container.

##### 3.1.2.2 Penetration Test for Hardness and Adhesiveness

The penetration hardness and adhesiveness tests were performed using a TA.XT2 Texture Analyzer (Texture Technologies Corp., Scarsdale, NY, USA; Stable Microsystems, Godalming, UK) as described by (Salunke and Metzger 2023) with some modifications. A 30 g, 10 mm thick sample of ice cream was placed on a petri plate immediately after manufacturing and stored at −20°C. A uniaxial penetration test was then conducted using a TA‐8 probe with a 6.35 mm diameter stainless steel ball, with the penetration depth set to 3 mm. All texture measurements were conducted at a controlled sample temperature of −20°C. All ice cream samples were stored at −20°C for 24 h prior to texture analysis.

##### 3.1.2.3 Melting Rate

The melting rate was determined as described by (Januário et al. 2018) with some modifications. To measure melting behavior, an ice cream sample was prepared using a scoop to form a half‐sphere block weighing 100 ± 1 g. The sample, initially at −15°C, was placed on a stainless‐steel wire screen (40 mesh) positioned over a funnel connected to a collection flask. The setup was transferred to a controlled temperature chamber maintained at 25°C, where all measurements were conducted. Drip loss was recorded every 15 min over a 3 h period by measuring the cumulative mass of melted ice cream collected in the flask. The melting rate (g/min) was calculated as the slope of the linear portion of the melting curve (melted mass vs. time) and used for statistical comparison among treatments. Although cumulative melted mass was recorded over time, only the calculated melting rate values are presented in Figure 9.

#### 3.1.3 Statistical Analyses

Data was analyzed using Costat 6.303 software (Cohort Software, Monterey, CA, USA) to evaluate differences among treatment means. A one‐way analysis of variance (ANOVA) under a completely randomized design was applied for parameters measured as single values, including rheological properties, overrun, texture, and melting rate. For the creaming index, a two‐way ANOVA was performed to evaluate the effects of treatment, storage time, and their interaction. When significant effects (*p* < 0.05) were observed, mean comparisons were conducted using the least significant difference (LSD) test. Replicates represent independent manufacturing batches, with each batch analyzed in duplicate or triplicate depending on the measurement.

## 4 Results and Discussion

### 4.1 Ice Cream Mix and Ice Cream Manufacture

During the ice cream mix preparation and manufacturing process, no significant challenges were encountered. However, notable differences were observed among the treatments, particularly in terms of viscosity and mix stability during the aging period. It was observed that both the IC sample and IC‐1 exhibited noticeably lower viscosity compared to the other treatments. This lower viscosity was associated with slight visible separation in the ice cream mix during the aging process, which was not observed in the other formulations. In contrast, the remaining treatments maintained a homogeneous consistency throughout the aging period, suggesting improved stability and potentially better emulsification or ingredient interaction. The pH values did not differ significantly (*p* > 0.05) among treatments (data not shown).

### 4.2 Ice Cream Mix

#### 4.2.1 Creaming Index Ice Cream Mix Samples

The creaming index (CI) serves as a key indicator of emulsion stability, with higher values indicating greater phase separation (Rozaini et al. 2024). It reflects the degree of fat globule separation from the aqueous phase during storage. An increase in CI indicates that more fat has risen to the top, forming a cream layer and suggesting reduced emulsion stability over time. Conversely, a lower CI indicates improved fat dispersion within the mix and greater emulsion stability (Cheng et al. 2015). As shown in Figure 2, all samples exhibited increasing CI over the 21 day storage period at 4°C. Two‐way ANOVA revealed a significant treatment × storage time interaction (*p* < 0.05), indicating that the effect of the different hyaluronic acid treatments on CI depended on the duration of storage, with the formulations exhibiting different rates of change over time.

**FIGURE 2:**
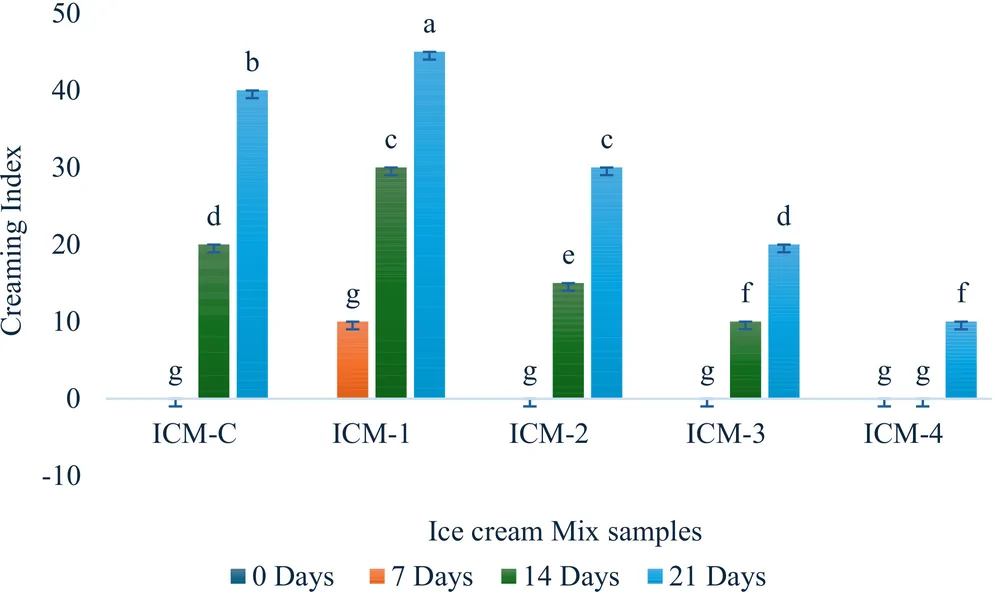
Creaming index of ice cream mix samples with different molecular weights of hyaluronic acid after 21 days of storage at 4°C.

The control (ICM‐C) and ICM1 (320 kDa HA) exhibited the highest CI values by Day 21, exceeding 40%, indicating greater instability during storage. In contrast, ICM3 (1550 kDa HA) and ICM4 (2550 kDa HA) maintained substantially lower CI values, with ICM4 showing minimal creaming even after 21 days. This behavior may be attributed to the higher water‐binding capacity and viscosity associated with higher molecular weight HA, which could contribute to improved resistance to phase separation; however, these mechanisms were not directly measured in the present study. These findings are consistent with Joshi et al. (2024), who reported that higher MW HA (980 kDa and 2550 kDa) improved emulsion properties and water‐holding capacity in skim milk systems. Similarly, Sutariya and Salunke (2023) observed that HA at 0.25% improved emulsifying activity and stability in milk systems. These results are further supported by studies on other dairy systems products (Alsaleem et al. 2024; Alsaleem and Hamouda 2024; Nie et al. 2022), which suggest that polysaccharides may enhance water retention and contribute to improved physical stability of the matrix.

#### 4.2.2 Rheological Analysis of Ice Cream Mix Samples

Understanding the rheological behavior of ice cream mixes is essential, as it influences texture, stability, and processing performance. This includes the storage modulus (G′), which represents the elastic or solid‐like behavior and reflects the material's ability to store energy; the loss modulus (G″), which indicates the viscous or liquid‐like behavior related to energy dissipation during deformation; and apparent viscosity, which describes the resistance of a fluid to shear (Hamouda and Salunke 2024). As shown in Figures 3 and 4, both the loss modulus (G″) and storage modulus (G′) increased with angular frequency, demonstrating typical viscoelastic behavior of the ice cream mixes. A clear positive relationship was observed between the molecular weight (MW) of HA and the values of G′ and G″. ICM‐4 (2550 kDa HA) recorded the highest moduli, followed by ICM‐3 (1550 kDa) and ICM‐2 (980 kDa), while ICM‐1 (320 kDa) and the control (ICM‐C) exhibited significantly lower values. Figure 5 also shows that as the MW of HA increased, the viscosity of the ice cream mixes increased. ICM‐4 exhibited the highest viscosity across the entire shear rate range, while the control mix (ICM‐C) showed the lowest values. This trend suggests that higher MW HA contributes to increased resistance to flow and enhanced viscoelastic behavior of the mixes. These effects may be associated with increased intermolecular interactions and structural organization within the system; however, these mechanisms were not directly measured in the present study. These findings are consistent with Joshi et al. (2024), who reported that increasing HA MW improved viscosity and rheological strength in milk systems. Similarly, Sutariya and Salunke (2022) observed that higher HA concentrations enhanced consistency and viscoelasticity. In agreement with these observations, Everett and McLeod (2005) demonstrated that the addition of non‐adsorbing polysaccharides such as guar gum and xanthan increased viscosity and viscoelastic moduli in milk systems, potentially through the formation of polymer‐rich phases that influence casein interactions. Similar trends have also been reported in other dairy systems, where polysaccharides such as xanthan gum increased viscosity and improved textural stability Alsaleem and Hamouda (2024). Overall, increasing the molecular weight of HA was associated with higher viscosity and viscoelastic moduli, which may contribute to improved structural stability and processing characteristics of ice cream mixes. These effects may be linked to enhanced water‐binding capacity and structural organization within the system; however, such mechanisms were not directly measured and should be considered as indicative rather than definitive. It is important to note that the control formulation included both stabilizers and emulsifiers, whereas the HA treatments did not. This difference may have influenced the observed rheological behavior, as emulsifiers are known to affect fat destabilization, air incorporation, and network formation in ice cream systems. Therefore, the improvements observed with HA should be interpreted within the context of this formulation difference.

**FIGURE 3:**
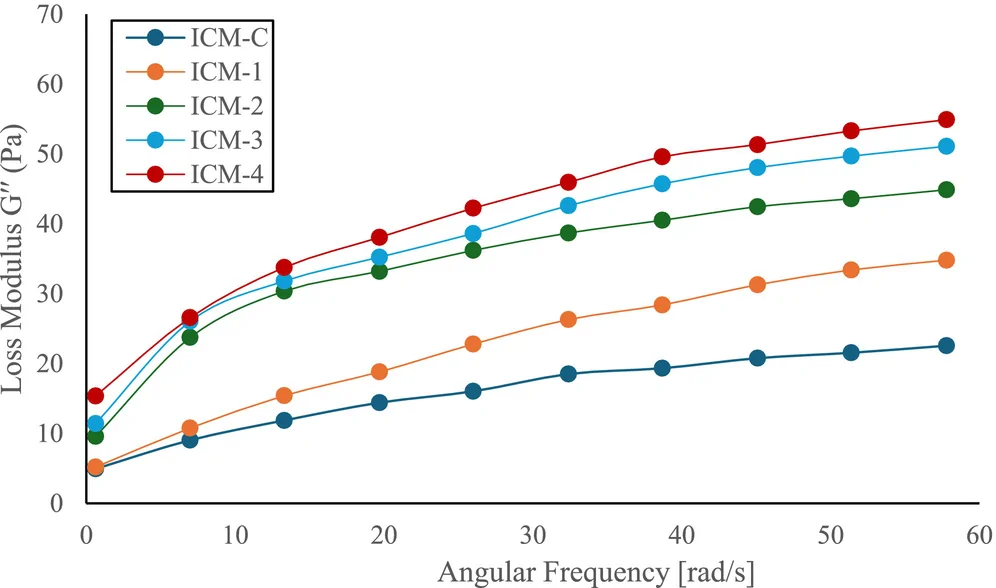
Effect of different molecular weights of hyaluronic acid on the loss modulus (G′′: Pa) of ice cream mix samples.

**FIGURE 4:**
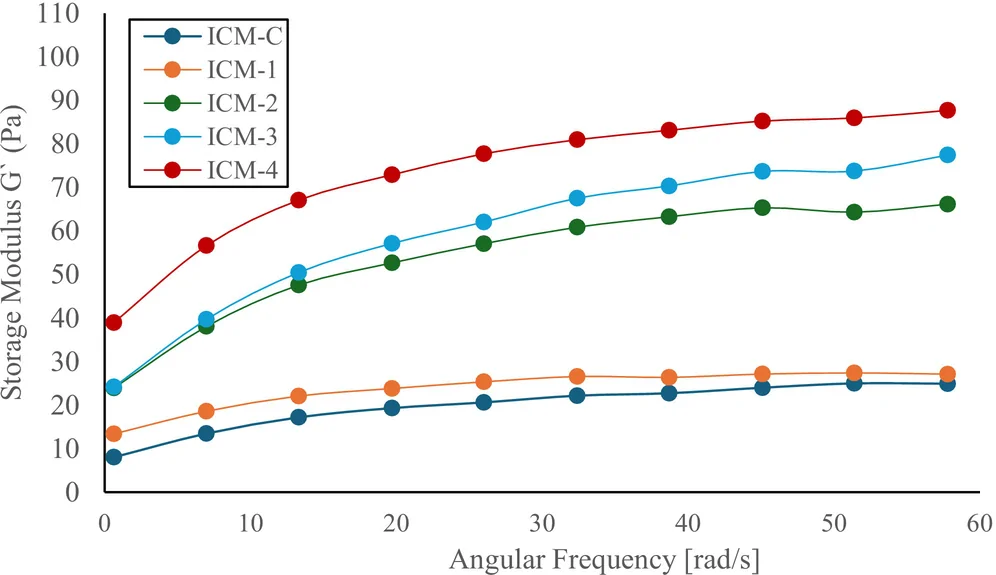
Effect of different molecular weights of hyaluronic acid on the storage modulus (G′: Pa) of ice cream mix samples.

**FIGURE 5:**
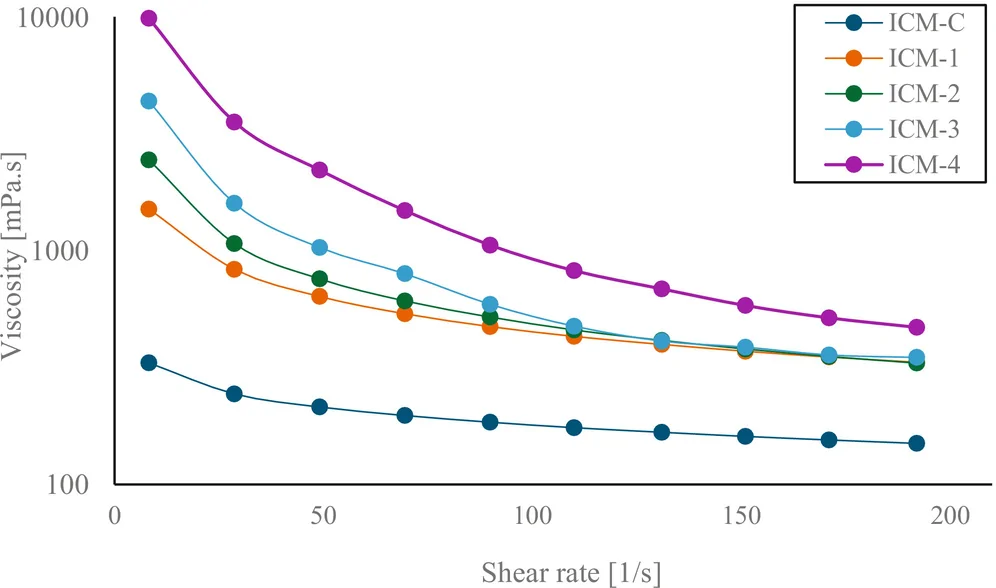
Effect of different molecular weights of hyaluronic acid on the viscosity [mPas] of ice cream mix samples.

### 4.3 Ice Cream Analysis

#### 4.3.1 Overrun

Overrun refers to the amount of air incorporated into ice cream during its production (Wu et al. 2019). It is expressed as a percentage representing the increase in volume resulting from air incorporation. This parameter is important as it influences the texture, taste, and appearance of the final product. Generally, higher overrun results in a lighter and softer ice cream, whereas lower overrun leads to a denser and creamier texture (Jana et al. 2016). The results presented in Figure 6 showed no significant difference (*p* > 0.05) among all samples, with values ranging from 29.20% for IC2 and IC3 to 30.86% for ICM‐C. The relatively low overrun observed across all samples may be attributed to the use of a small‐scale batch ice cream freezer, which can limit air incorporation efficiency. The results suggest that the addition of HA did not significantly influence overrun under the conditions of this study. The similarity in overrun values between HA‐treated samples and the control indicates that HA incorporation did not adversely affect air incorporation. These findings are consistent with Yuennan et al. (2014), who reported that ice cream systems containing hydrocolloids such as guar gum or okra cell wall exhibited lower overrun compared to control samples without stabilizers. This behavior may be related to increased viscosity, which could reduce the efficiency of air incorporation during freezing; however, this mechanism was not directly evaluated in the present study.

**FIGURE 6:**
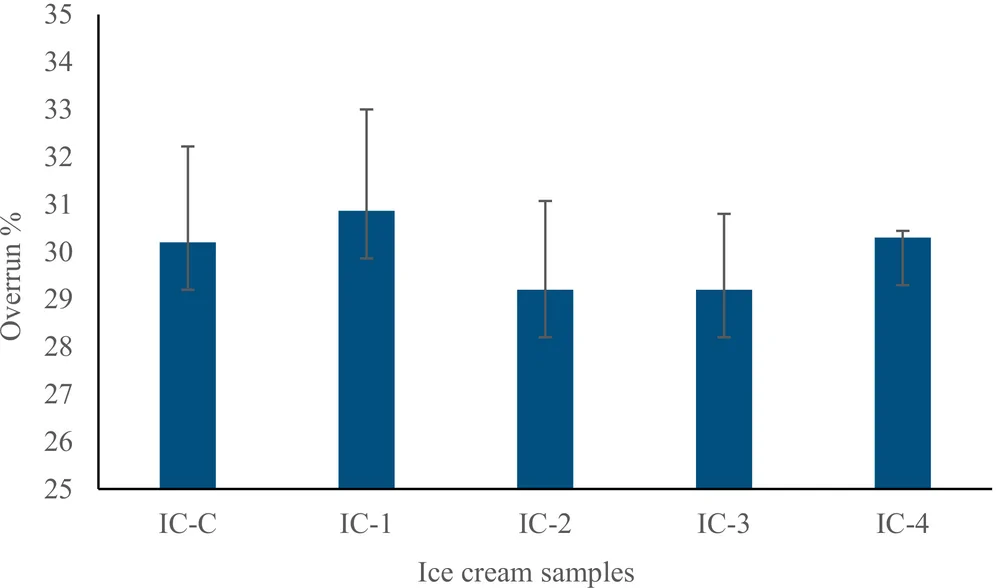
Effect of different molecular weights of hyaluronic acid on the overrun of ice cream samples.

#### 4.3.2 Penetration Test for Hardness and Adhesiveness of Ice Cream Samples

Hardness is a key quality attribute in ice cream, as it affects scoopability, mouthfeel, and overall consumer acceptance. Optimal hardness helps maintain product structure during storage and contributes to resistance against rapid melting. It also influences the sensory experience by balancing firmness and smoothness to achieve a desirable texture (Kwak et al. 2016). The results presented in Figure 7 indicate a significant effect (*p* < 0.05) of HA molecular weight (MW) on the hardness of ice cream samples. The control sample (IC‐C), which contained a traditional stabilizer, and IC‐1 (320 kDa HA) exhibited significantly lower hardness values than IC‐2, IC‐3, and IC‐4, which incorporated higher‐MW HA (980, 1550, and 2550 kDa, respectively).

**FIGURE 7:**
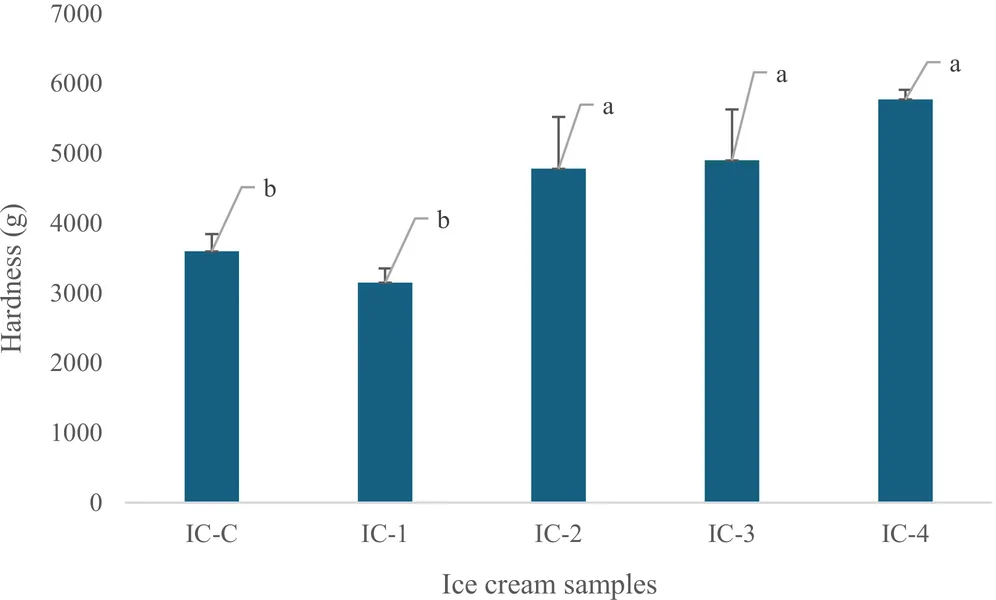
Effect of different molecular weights of hyaluronic acid on the hardness of ice cream samples.

This behavior may be associated with the higher water‐binding capacity and viscosity of higher MW HA (Joshi et al. 2024), which could contribute to increased resistance to deformation. It is also possible that higher MW HA promotes greater structural organization within the matrix; however, these mechanisms, including potential hydrogen bonding interactions and effects on ice crystal mobility, were not directly measured in the present study. Joshi et al. (2024) reported that increasing HA MW enhanced the water‐holding capacity of skim milk systems. In the present study, IC‐2, IC‐3, and IC‐4 showed no significant differences among themselves but were significantly harder than the control and IC‐1, suggesting that HA with an MW ≥ 980 kDa is associated with increased firmness. Similar trends have been observed in other dairy systems; for example, Jiao et al. (2025) reported increased hardness in Mozzarella cheese upon incorporation of high‐MW HA, attributed to enhanced structural interactions. As shown in Figure 8, all samples exhibited negative adhesiveness values, indicating cohesive behavior. Although IC‐2 showed the highest adhesiveness, no significant differences (*p* > 0.05) were observed among treatments, suggesting that HA molecular weight did not substantially affect adhesiveness under the conditions of this study.

**FIGURE 8:**
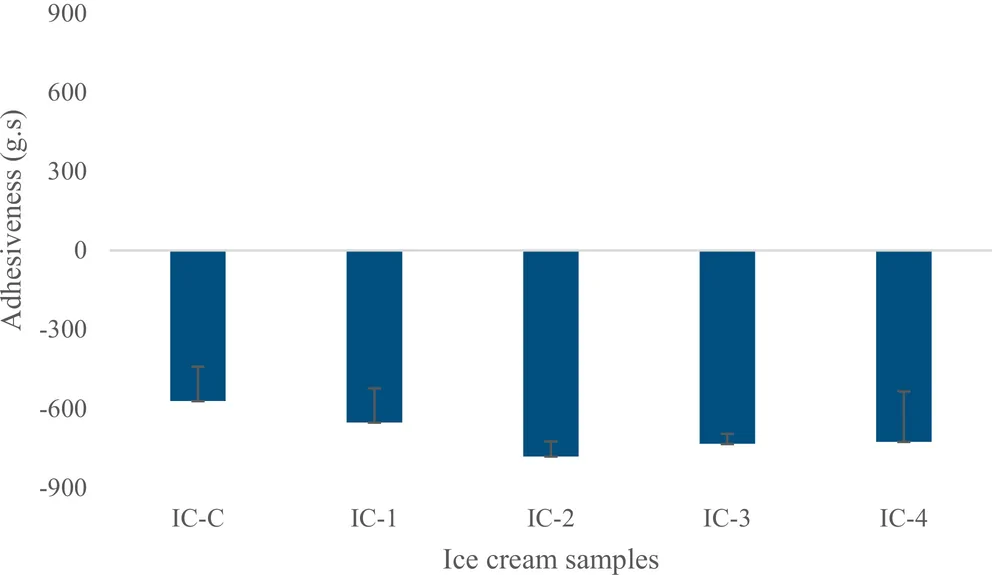
Effect of different molecular weights of hyaluronic acid on the adhesiveness of ice cream samples.

#### 4.3.3 Melting Rate

The melting rate of ice cream refers to the rate at which the product transitions from a solid to a liquid state under ambient conditions and is an important indicator of structural stability and product quality (Pei and Schmidt 2010). Melting rate is an important quality attribute of ice cream, as it influences product stability during serving and contributes to the overall consumption experience. The desired melting behavior depends on the product formulation and intended product characteristics (Jana et al. 2016). As shown in Figure 9, samples containing higher MW HA, particularly IC‐4 (2550 kDa) and IC‐3 (1550 kDa), exhibited significantly lower melting rates compared to IC‐1 (320 kDa) and the control (IC‐C). This behavior may be associated with the higher viscosity and water‐binding capacity of high MW HA (Joshi et al. 2024; Sutariya and Salunke 2022), which could contribute to improved resistance to structural breakdown during melting. However, these mechanisms were not directly measured and should be considered as hypotheses rather than confirmed effects. Similar trends have been reported by Liu et al. (2023), who observed reduced melting rates in ice cream systems containing polysaccharides, particularly in samples with higher serum phase viscosity.

**FIGURE 9:**
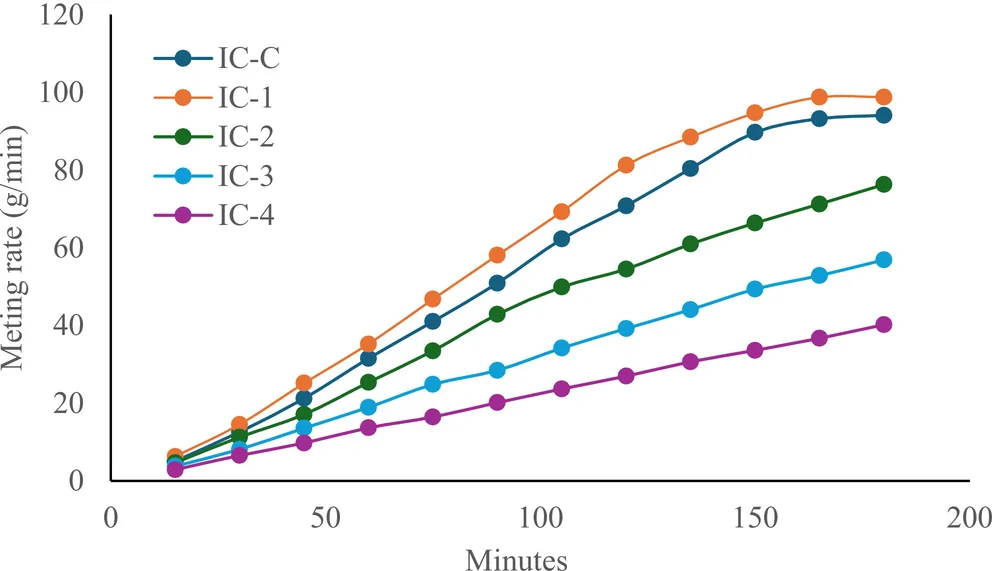
Effect of different molecular weights of hyaluronic acid on the melting rate (g/min) of ice cream samples.

## 5 General Outcomes

This study demonstrated that incorporating HA with varying molecular weights can significantly influence the physicochemical and rheological properties of ice cream and ice cream mix. While HA had no significant impact on overrun, it positively affected key quality attributes, including hardness, melting rate, creaming stability, and rheological behavior. Higher MW HA (≥ 980 kDa), especially 2550 kDa, resulted in increased hardness and viscosity, reduced melting rates, and lower creaming index values, indicating improved structural integrity, thermal resistance, and emulsion stability. The rheological analysis confirmed a strong positive correlation between HA MW and both storage and loss moduli, as well as apparent viscosity, indicating a more stable and cohesive matrix in high‐MW HA treatments.

A key limitation of this study is the difference in formulation between the control and HA‐treated samples. The control contained a stabilizer–emulsifier blend, while HA treatments were formulated without added emulsifiers. As a result, the comparison does not isolate the effect of stabilizer type alone, and the observed differences may partially arise from the absence of emulsifiers in the HA systems. Consequently, the findings should not be interpreted as a direct equivalence or full replacement of conventional stabilizer–emulsifier systems. Further studies incorporating matched emulsifier systems, along with microstructural and sensory analyses, are required to establish the full replacement potential of HA.

## 6 Conclusion

This study demonstrated that hyaluronic acid (HA) influences the physicochemical and rheological properties of ice cream depending on its molecular weight. Although no significant effect on overrun was observed, higher molecular weight HA (≥ 980 kDa) was associated with increased hardness, reduced melting rate, improved creaming stability, and enhanced rheological properties. High molecular‐weight HA (particularly 1550 and 2550 kDa) improved several measured properties of the tested ice cream formulations and may have potential as a structuring ingredient or partial alternative in frozen dairy dessert systems. However, as the control formulation contained both stabilizers and emulsifiers, these results do not demonstrate full replacement of conventional stabilizer–emulsifier systems. Further studies incorporating equivalent formulation systems and additional structural analyses are required to better understand the role of HA in ice cream applications.

## Author Contributions

**Rutvi Joshi:** conceptualization, methodology, data curation, investigation, validation, formal analysis, writing – original draft, project administration, writing – review and editing. **Mahmoud E. A. Hamouda:** conceptualization, investigation, methodology, validation, formal analysis, data curation, writing – original draft, writing – review and editing. **Prafulla Salunke:** conceptualization, methodology, investigation, validation, supervision, funding acquisition, visualization, project administration, resources, writing – review and editing.

## Funding

The authors have nothing to report.

## Conflicts of Interest

The authors declare no conflicts of interest.

## Data Availability

The data that support the findings of this study are available from the corresponding author upon reasonable request.
